# Effects of novel pyrrolomycin MP1 in MYCN amplified chemoresistant neuroblastoma cell lines alone and combined with temsirolimus

**DOI:** 10.1186/s12885-019-6033-2

**Published:** 2019-08-27

**Authors:** Timothy R. McGuire, Don W. Coulter, Dachang Bai, Jason A. Sughroue, Jerry Li, Zunhua Yang, Zhen Qiao, Yan Liu, Daryl J. Murry, Yashpal S. Chhonker, Erin M. McIntyre, Gracey Alexander, John G. Sharp, Rongshi Li

**Affiliations:** 1 Department of Pharmacy Practice and Science, College of Pharmacy, University of Nebraska Medical Center, 986145 Nebraska Medical Center, Omaha, NE 68198-6145 USA; 2 College of Medicine, Division of Pediatrics, University of Nebraska Medical Center, Omaha, NE USA; 3 Genetics, Cell Biology and Anatomy, University of Nebraska Medical Center, Omaha, NE USA; 4 Chemistry and Medicinal Chemistry, UNMC Center for Drug Discovery & Department of Pharmaceutical Sciences, 986125 Nebraska Medical Center, Omaha, NE 68198 USA

**Keywords:** Pyrrolomycin Marinopyrrole (MP1), Temsirolimus, Neuroblastoma, MYCN, Metabolism, Mitochondria

## Abstract

**Background:**

The activity of MP1, a pyrrolomycin, was studied in MYCN amplified neuroblastoma (NB) alone and combined with temsirolimus (TEM).

**Methods:**

Activity of MP1 was tested in MYCN amplified (BE-2c, IMR) and non amplified (SKN-AS) NB cells. The effect of MP1 on MYCN, MCL-1, cleaved PARP, LC3II/LC3I, bcl-2, BAX, and BRD-4 were determined by western blot and RNAseq. The effect of MP1 on metabolism, mitochondrial morphology, and cell cycle was determined. Toxicology and efficacy of MP1 plus TEM were evaluated.

**Results:**

The IC_50_ of MP1 was 0.096 μM in BE-2c cells compared to 0.89 μM in IMR, and >50 μM in SKN-AS. The IC_50_ of MP1 plus TEM in BE-2c cells was 0.023 μM. MP1 inhibited metabolism leading to quiescence and produced a decline in cell cycle S-phase. Electron microscopy showed cristae loss and rounding up of mitochondria. Gene and protein expression for MYCN and MCL-1 declined while LCII and cleaved PARP increased. Protein expression of BAX, bcl-2, and BRD-4 were not significantly changed after MP1 treatment. The in-vivo concentrations of MP1 in blood and tumor were sufficient to produce the biologic effects seen in-vitro. MP1 plus TEM produced a complete response in 3 out of 5 tumor bearing mice. In a second mouse study, the combination of MP1 and TEM slowed tumor growth compared to control.

**Conclusions:**

MP1 has a potent inhibitory effect on the viability of MYCN amplified NB. Inhibition of metabolism by MP1 induced quiescence and autophagy with a favorable toxicology and drug distribution profile. When combined with TEM anti-tumor activity was potentiated in-vitro and in-vivo.

**Electronic supplementary material:**

The online version of this article (10.1186/s12885-019-6033-2) contains supplementary material, which is available to authorized users.

## Background

Neuroblastoma (NB) is a rare childhood tumor with about 700 new cases per year in North America [1]. Prognosis is related to age at diagnosis, histology, and amplification of the oncogene, MYCN [2]. MYCN is commonly amplified in high-risk NB and is linked to NB cell metabolism supporting oxidative glycolysis or Warburg metabolism [3, 4]. Warburg metabolism may be linked to a loss of functional mitochondrial mass through mitophagy or from intrinsic abnormalities in cancer cell mitochondria [4, 5]. MYCN amplified cancer cells also depend on mitochondrial metabolism to supply Krebs Cycle intermediates and the high energy demands associated with MYCN induced proliferation [6]. The difference in metabolism between cancer cells and normal cells is increasingly being targeted therapeutically [4–7]. Since MYCN amplification can activate both glycolysis and oxidative phosphorylation (OXPHOS), inhibition of MYCN pathways may produce a severe decline in metabolic intermediates and ATP leading to autophagy, quiescence, and cell death.

We previously reported marinopyrroles as active antibiotic and anticancer agents [8–11]. In our ongoing program to improve physicochemical and drug-like properties of marinopyrroles, we designed a novel series of pyrrolomycin-based natural product derivatives. In this study, a MYCN amplified NB cell line, BE-2c, was used to determine activity of the pyrrolomycins in an in-vitro screening assay. The most active pyrrolomycin (MP1) was identified and potential mechanisms of activity were studied. From the activity seen in the screening experiments we hypothesized that MP1 was inhibiting the tumor driver and oncogene, MCYN. MCL-1 inhibition was reported previously by our group using the related compound marinopyrrole A in leukemia cell lines [12].

MYC amplification leads to stimulation of a number of pathways involved in cancer progression and therapy resistance. One of the major pathways stimulated in MYC amplified tumors is the PI3K-AKT-mTOR pathway. It was this association between MYCN amplification and the mTOR pathway along with the increasing use of the mTOR inhibitor temsirolimus (TEM) in clinical trials to treat NB that led to experiments combining TEM with MP1 reported in this manuscript.

## Methods

The Pediatric Cancer Research Laboratory at University of Nebraska Medical Center in concert with the drug development laboratory of Dr. Rongshi Li, undertook pre-clinical studies to determine the activity and potential mechanism of action of the marinopyrrole MP1. The combination of viability assays, cell cycle analysis, cellular ultra-structure, metabolic flux analysis, western blots, and RNAseq were used to study the effect of MP1 alone and combined with TEM. These in-vitro studies informed two in-vivo experiments, an initial small pilot to assess toxicity of MP1 plus TEM and a second activity study of each agent alone and in combination. All in-vitro and in-vivo experiments integrated vehicle controls for comparison to treatment groups.

### A library of natural product-based small molecules

**Fig. 1:**
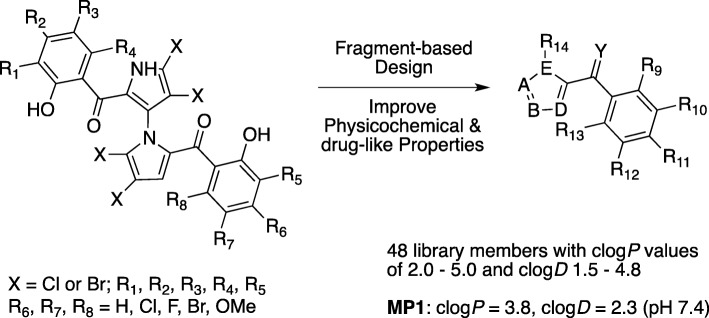
A “Magic” library of natural product derivatives from fragment-based and structural optimization of marinopyrroles. MP1 has physicochemical properties which are acceptable for drug development with cLog*P* = 3.8, a value which indicates a moderate hydrophilicity measured by logarithm of octanol/water partition coefficient. cLogD = 2.3 which integrates pH into the calculation is also acceptable for drug development at physiologic pH (7.4)

Marinopyrroles have poor water solubility with clog*P* values up to 6.5 which is too high and not ideal solubility for drug development. In order to improve their physicochemical and drug-like properties, we designed and synthesized a library containing 48 members with lower clog*P* values ranging from 2.0 to 5.0. MP1 was one of these derivatives with a clog*P* value of 3.8 (clog*D* 2.3 at pH 7.4). MP1 was fully characterized using ^1^H and ^13^C NMR and high resolution Mass Spectroscopy after reverse phase HPLC purification (Fig. 1). Purity was required to be greater than 99% prior to determining in-vitro and in-vivo activity.

### Cell lines

BE-2c, a MYCN amplified NB cell line (ATCC: CRL-2268), was used to model high-risk chemoresistant NB. A 1:1 mixture of EMEM and F12 medium along with 10% FBS was used to grow BE-2-c cells. Cells were passaged at a 1:4 ratio and media renewed every 3 days. All experiments were performed using cells that were 70–80% confluent. While BE-2c cells were used in the majority of the experiments, the activity of MP1 was also studied in the MYCN amplified cell line, IMR (CCL-127) and one non-MYCN amplified cell-line SKN-AS (CRL-2137). Cell lines were verified for identity using ATCC Cell Authentication Service using short tandem repeat profiling.

### 3-(4,5-Dimethylthiazol-2yl)-2,5-Diphenyltetrazolium (MTT) assay

BE-2c, IMR-32, and SKN-AS cells were seeded at a density of 25,000–40,000 cells per well of a 96 well plate. Initial screening experiments were performed on 19 marinopyrroles of the MP series. MP1 was the most active marinopyrrole in BE-2c cells. BE-2c cells were treated with MP1 at concentrations of 0.1, 0.25, 0.50, 1.0, 5.0, 10.0, and 50.0 μM for 18 h and IC_50_ calculated. MP1 was diluted in DMSO and each 96 well plate included media only controls and DMSO plus media controls. Ten microliters of MTT (5 mg/ml) solution was added to each well and after a 4 h incubation at 37 °C well contents were solubilized and absorption measured at 550 nm. The average absorbance of DMSO controls was used to calculate a percentage of control which was regressed (non-linear) against the concentration of MP1 and the IC_50_ calculated (Graph Pad Prism, version 6.02, LaJolla, CA). The addition of TEM at a concentration of 1 μM was added to the MP1 concentration scheme to evaluate potentiation.

### Flow cytometry using propidium iodide staining: cell cycle analysis/apoptosis

BE-2c cells were treated for 18 h with MP1 at a concentration of 500 nM. Cells were passaged using trypsin-EDTA 0.25% and counted. One million cells were washed with 1X PBS and fixed with 400uL ice cold 1x PBS plus 800uL ice cold 100% EtOH and stored at 4 °C for at least 2 h. Cells were equilibrated to 25 °C, spun, and further washed with 1x PBS. Using a solution of 400uL 1x PBS, 1x Propidium Iodide (PI), plus 1x RNase, cells were incubated at 37 °C for 30 min and placed on ice for analysis. PI stained samples were run on a YETI Flow Cytometer (Propel Labs) with 561 nm excitation and fluorescence emission and read in the 615/24 nm channel. Single cells were gated based on fluorescence width versus fluorescence height signals and cell cycle analysis was performed on the PI-area signal from single cells using ModFit Software (Verity Software House, Topsham, Maine).

### Western blots analysis in BE-2c cells

A number of proteins were analyzed to evaluate the potential mechanism of action of MP1 including modulators of apoptosis (cleaved PARP, BAX, and bcl-2), autophagy (LC3II/I), BRD-4, and MCL-1 and MYCN oncogenes. All primary antibodies were either mouse or rabbit (Cell Signaling, Danvers, MA) with complimentary mouse or rabbit secondary antibodies (AbCam, Cambridge, MA). Total proteins were isolated from BE-2c cells using RIPA lysis buffer and quantified using the BCA assay. Protein was loaded (20 mcg) and resolved on precast polyacrylamide gels and transferred onto nitrocellulose membranes. The primary antibodies for each of the proteins listed above were used at a dilution of 1:1000 per manufacturer’s recommendations. Beta-actin, cyclophilin, and total protein served as a loading controls. IgG secondary antibodies were used at a dilution of 1:2000. Detection was performed using a MyECL Imager (ThermoScientific, MA, USA) and band density was normalized using loading controls.

### Determination of MYCN gene copy number using digital PCR

The MYCN copy number of the three cell lines was confirmed using digital PCR. Briefly, BE-2c, IMR and SKN-AS cells were seeded in a 6 well plate at a density of 300,000 cells per well and allowed to grow overnight. DNA was isolated using Qiagen Blood and Tissues kit. DNA concentration was measured using Nano- Drop 2000c Spectrophotometer (ThermoFisher Scientific, Waltham, MA). The QX200 Droplet Digital PCR System (Bio-Rad Laboratories, Munich, Germany) was used to detect *MYCN.*

### RNA isolation and RNAseq and gene expression analysis

Total RNA was extracted using the RNeasy Micro Kit (Qiagen, Germantown, MD). Nano Drop was used to measure RNA concentration and purity and integrity was evaluated using Agilent Bioanalyzer System (Agilent Technologies, Santa Clara, CA). RNA sequencing and library preparation was performed using Maestro TruSeq Application (Perkin Elmer, Waltham, MA). Parameters used were optimized for 50 bp single-end reads with 20 million reads per sample. Three independent samples were analyzed and reported for control, MP1, TEM, and the combination. Gene expression analysis are reported for those pathways where protein analysis was performed (MYCN, MCL-1, PARP, LC3I, LC3II, BRD-4, bcl-2, and BAX).

### Metabolic profiles associated with MP1 treatment

Metabolic flux analysis using the XFp Seahorse® Metabolic Analyzer (Agilent) which measures both OXPHOS and glycolysis using the combination of oxygen consumption rate (OCR) for OXPHOS and extracellular acidification rate (ECAR) for glycolysis was used to measure the effect of MP1 treatment on BE-2c cell metabolism.

### Metabolic phenotype associated with MP1 treatment

Six wells of an XFp culture plate were seeded with 15,000 BE-2c cells/well in 80 μL DMEM with 10% FBS. Cells were incubated for 6 h @ 37 °C/5% CO_2_ before drug treatments: 3 wells were treated with 0.01% DMSO and 3 wells with MP1 at varying concentrations (100 nM, 200 nM, 500 nM, and 750 nM). After 18 h of MP1 treatment, cells were washed with XF base media supplemented with 10 mM glucose, 1 mM sodium pyruvate, and 2 mM L-glutamine and incubated at 37 °C for 1 h prior to assay. The XFp Cell Energy Phenotype assay utilizes oligomycin as an inhibitor of ATP synthase and FCCP which is a mitochondrial uncoupling reagent. Oligomycin inhibits mitochondrial ATP production leading to a compensatory increase in glycolysis to meet energy demands. FCCP depolarizes the mitochondrial membrane driving the oxygen consumption rates higher as mitochondria restore polarization. The results were graphed on a grid indicating degree of aerobic/glycolytic metabolism and energetic/quiescent phenotypes.

### Transmission electron microscopy (EM)

EM was performed on BE-2c cells treated for 18 h with MP1 and compared to no treatment controls. Samples for EM imaging were fixed by immersion in a solution of 2% glutaraldehyde, 2% paraformaldehyde in a 0.1 M Sorenson’s phosphate buffer (pH 6.2) for a minimum of 24 h at 4 °C. During processing, samples were post-fixed in a 1% aqueous solution of osmium tetroxide for 60 min. Subsequently, samples were dehydrated in a graded ethanol series and propylene oxide was used as a transition solvent between the ethanol and araldite resin. Samples were allowed to sit overnight in a 1:1 mixture of propylene oxide:resin allowing all the propylene oxide to evaporate. Samples were then incubated in fresh resin for 2 h at room temperature before final embedding. Thin sections (100 nm) made with Leica UC6 Ultra microtome were placed on 200 mesh copper grids, and stained with 2% Uranyl Acetate, followed by Reynolds Lead Citrate. Grids were examined on a Tecnai G2 Spirit *TWIN* (FEI) operating at 80 kV and *images* were acquired digitally with an AMT imaging system.

### Treatment of tumor bearing NSG mice with MP1 alone and combined with TEM

The animal experiments were approved by the UNMC IACUC (protocol#: 13–050-00-Fc). Female NSG (20–25 g) mice between the ages of 8–10 weeks were used to test for MP1 anti-tumor activity, toxicity, and MP1 concentrations in blood and tumor. Mice were euthanized by CO_2_ at an initial flow rate of 10–20% of chamber volume per minute and once unconscious the flow rate was increased to speed the time to death. After CO_2_ euthanasia, cervical dislocation was used as a physical secondary method to ensure death. NSG mice were injected subcutaneously with 5 × 10^5^ BE2-c cells in a 50:50 PBS/Matrigel® solution. In a tolerability study, 6 mice received MP1 alone at a dose of 15 mg/kg/day five times per week by oral gavage for 10 doses. Blood was collected at necropsy for evaluation of hematologic parameters (WBC, RBC, HgB, and platelets) and liver, spleen, and brain were examined histologically for signs of toxicity. Bone marrow was collected at necropsy for a CFU-GM assay to assess bone marrow toxicity. Drug concentration of MP1 in blood and tumor were performed using an LC-MS-MS assay to characterize MP1 concentrations achieved in blood and tumor.

The initial assessment of combination therapy used 5 mice testing the combination of MP1 (15 mg/kg orally 5x per week) and TEM (10 mg/kg IP 5x per week). A follow up study of the combination integrated control groups and modified dosing of MP1 plus TEM to three times per week at the doses described above. Groups included diluent control (*N* = 10), MP1 alone (*N* = 5), TEM alone (*N* = 5), and the combination (*N* = 5). Tumor measurements were performed daily and treatments began on the first day the tumor achieved 2 mm^3^ in size.

### LC-MS/MS conditions for MP1 quantitation

A Shimadzu LC-MS/MS system (LC-MS/MS 8060, Shimadzu, Japan) was used for quantitative estimation of MP1. Mass spectrometric detection was performed using a DUIS source in negative electrospray ionization mode. The MS/MS system was operated at unit resolution in the multiple reaction monitoring mode, using precursor ion>product ion combinations of 324.10 > 168.30 m/z for MP1 and 411.95 > 224.15 m/z for PL-3, used as an internal standard. UPLC and MS systems were controlled by LabSolutions LCMS Ver. 5.6 (Shimadzu Scientific, Inc.). The compound MP1 resolution and acceptable peak shape was achieved on an Acquity UPLC BEH C18 column (1.7 μm, 100 × 2.1 mm, Waters, Inc. Milford MA) protected with a C18 guard column (Phenomenex, Torrance CA). Mobile phase consisted of 0.1% acetic acid in water (mobile phase A) and methanol (mobile phase B), at total flow rate of 0.25 ml/min. The chromatographic separation was achieved using isocratic elution over 6 min. The injection volume of all samples was 10 μl. The assay was linear over the range of 0.1 to 500 ng/ml.

### Biodistribution of MP1

The biodistribution of MP1 was evaluated in NSG mice administered at a dose of 15 mg/kg five times per week via oral gavage. The animals were euthanized and blood, organs and tumor harvested at 0.5, and 24 h post-administration and stored at − 80 °C. Tissues and tumor were homogenized in water prior to sample preparation. The calibration and quality control samples were separately prepared for MP1 by spiking 10 μl of appropriate calibration stock of MP1, in 100 μl of blank biometrix to obtain a concentration range of 0.5–500 ng/ml and 10 μl of internal standard solution (1.0 μg/ml). For the study sample, 25 μl of plasma or 100 μl of tissue homogenate were used. Ice-cold concentrated acetonitrile (600 μl) was added to each sample to initiate protein precipitation. The mixture was vortexed for 2 min, followed by centrifugation at 17,950 x g for 20 min at 4 °C.

### Statistical analysis

Student’s T-test for unpaired data was used in two group comparison of normally distributed data and One-Way Analysis of Variance for multiple groups. For non-parametric comparisons Mann-Whitney Rank Sum and Kruskall Wallis were used. Normality was tested using D’Agostino and Pearson Test for Normality. Statistical significance was defined as *p* ≤ 0.05.

## Results

### Effect of MP1 on BE-2c, IMR, and SKN-AS viability

**Fig. 2:**
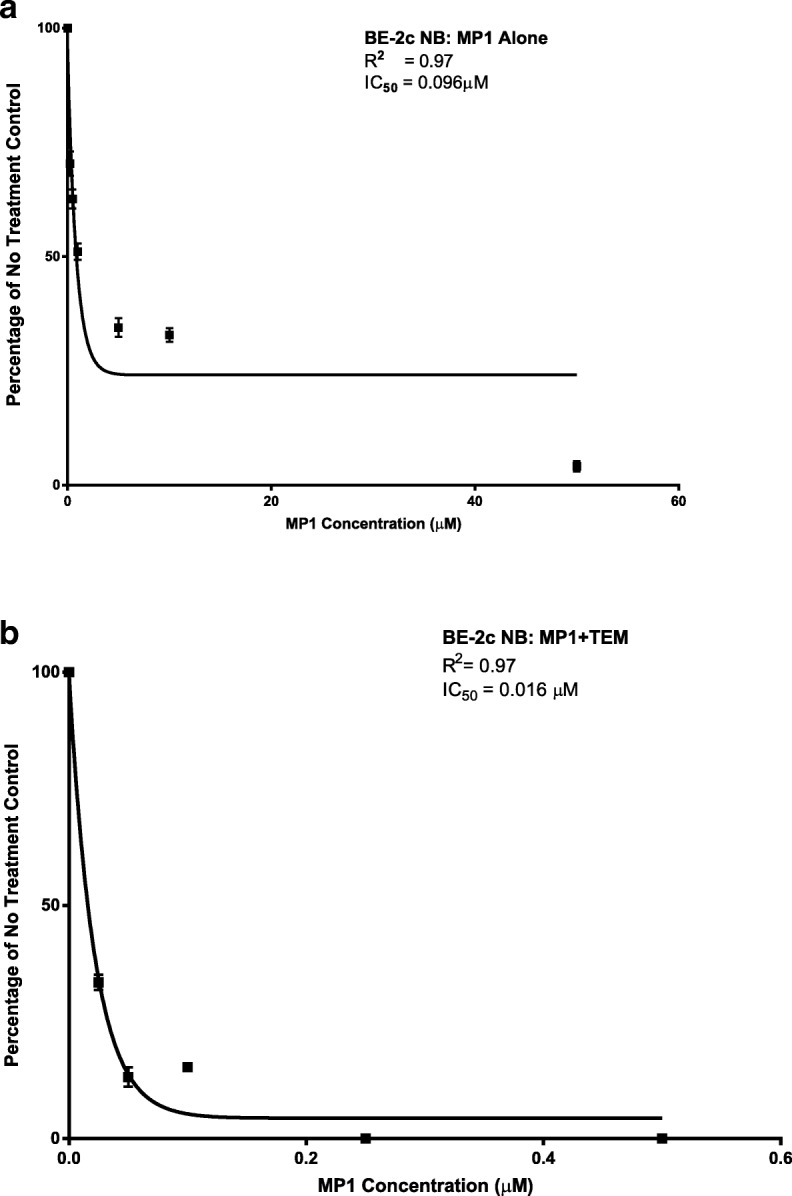
**a** Activity of MP1 alone in highly MYCN amplified NB cell line BE-2c. Treatments were for 18 h at concentrations of MP1 of 0.1 μM, 0.25 μM, 0.5 μM, 1.0 μM, 5.0 μM, 10.0 μM, and 50 μM **b** MP1 plus TEM at MP1 concentrations of 0.025 μM, 0.050 μM, 0.1 μM, 0.25 μM, and 0.5 μM plus 1.0 μM. Reported as means of 8 data points ± SD

Figure 2a,b shows the concentration effect of MP1 alone and combined with TEM in BE-2c cells. BE-2c cells were highly sensitive to MP1 alone and when combined with TEM with an IC_50_ of 0.096 μM and 0.016, respectively. SKN-AS cells are MYCN non-amplified and were resistant to MP1 with IC_50_ > 50 μM. IMR which are intermediately MYCN amplified and were intermediately sensitive with an IC_50_ value of 0.88 μM (Additional file 1: Figure S1a,b). Additional file 2: Figure S2 confirms the relative expression of MYCN in BE-2c, IMR-32, and SKN-AS cells by droplet digital PCR and confirms previous data from the literature [13, 14].

### MP1 effects on cell cycle in BE-2c cells

Table 1 reports the percentage of cells in the various phases of the cell cycle with and without treatment with MP1. The effects of MP1 on the cell cycle suggests a concentration dependent decline in the S-phase fraction and a concentration dependent increase in G2-phase cells, consistent with an anti-proliferative effect of MP1 on BE-2c cells.

**Table 1:** Cell cycle analysis in BE-2c cells using propidium iodide flow cytometry. Performed after 18 h of pre-treatment with MP1 at varying concentrations. Controls included media plus cells and vehicle

Group	%G1 Phase (SD)	%S Phase (SD)	%G2 Phase (SD)
Media Control	46.5 (5.2)	44.8 (4.6)	8.8 (1.7)
Vehicle	48.8 (5.6)	43.0 (5.9)	9.3 (1.5)
MP1 (0.25 μM)	64.5 (3.8)	28.8 (3.6)	6.8 (0.96)
MP1 (0.50 μM)	62.8 (6.7)	27.8 (3.1)	9.5 (3.7)
MP1 (1.0 μM)	50.3 (8.3)	31.3 (4.2)	18.3 (6.0)
MP1 (2.5 μM)	48.0 (7.4)	32.5 (5.1)	19.5 (6.9)
MP1 (5.0 μM)	48.5 (7.0)	32.8 (2.2)	18.0 (6.5)

### Metabolic effects of MP1 on BE-2c neuroblastoma cells

**Fig. 3:**
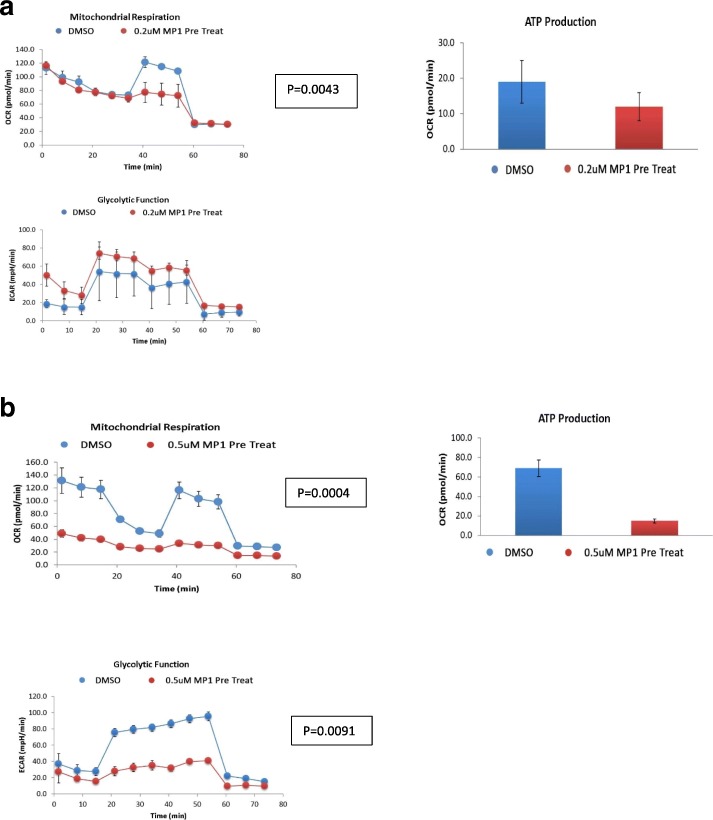
**a** Glycolysis, OXPHOS metabolism and ATP production after 0.2 μM MP1 **b** glycolysis and OXPHOS metabolism and ATP production after 0.50 μM of MP1. Blue line is DMSO control and the red line MP1 treated cells for 18 h, reported as mean ± SD. OXPHOS and glycolysis were measured by oxygen consumption rate (OCR) and extracellular acidification rate (ECAR), respectively. The shape of the OCR and ECAR curves result from the sequential treatment of cells with activators and inhibitors of OXPHOS and glycolysis. Statistical significance defined as *p* < 0.05

**Fig. 4:**
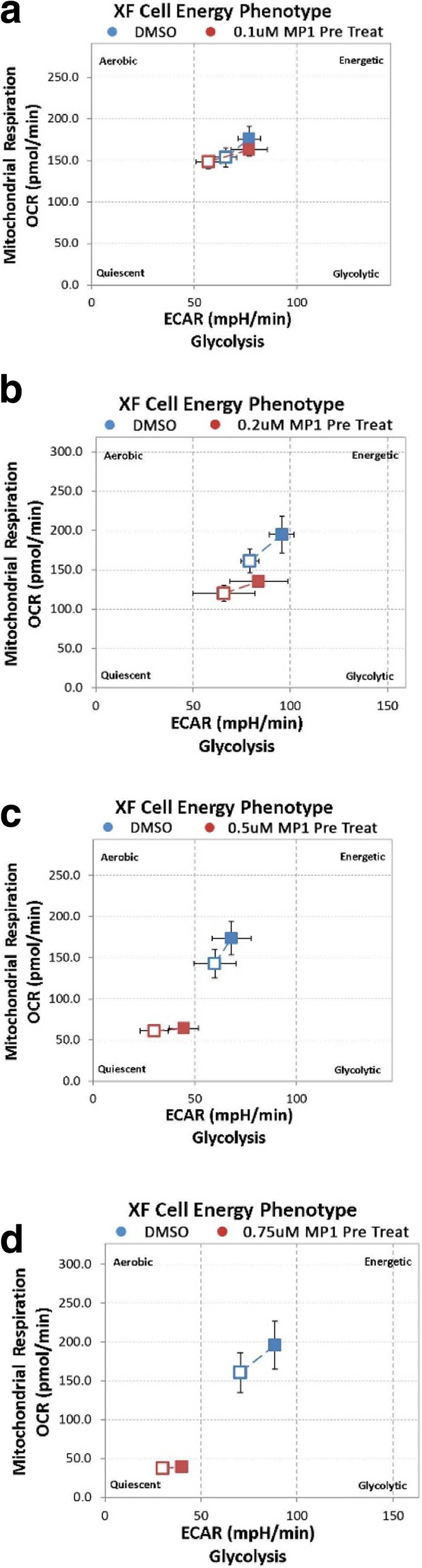
Metabolic phenotype after 18 h treatment with MP1. Open squares indicate unstressed and solid squares, stressed cells. Stress was induced using oligomycin as an ATP synthase inhibitor and FCCP which depolarizes the mitochondrial membrane. Blue is DMSO control and red is MP1 both after 18 h treatment at: **a** 0.1 μM **b** 0.2 μM **c** 0.5 μM and **d** 0.75 μM

**Fig. 5:**
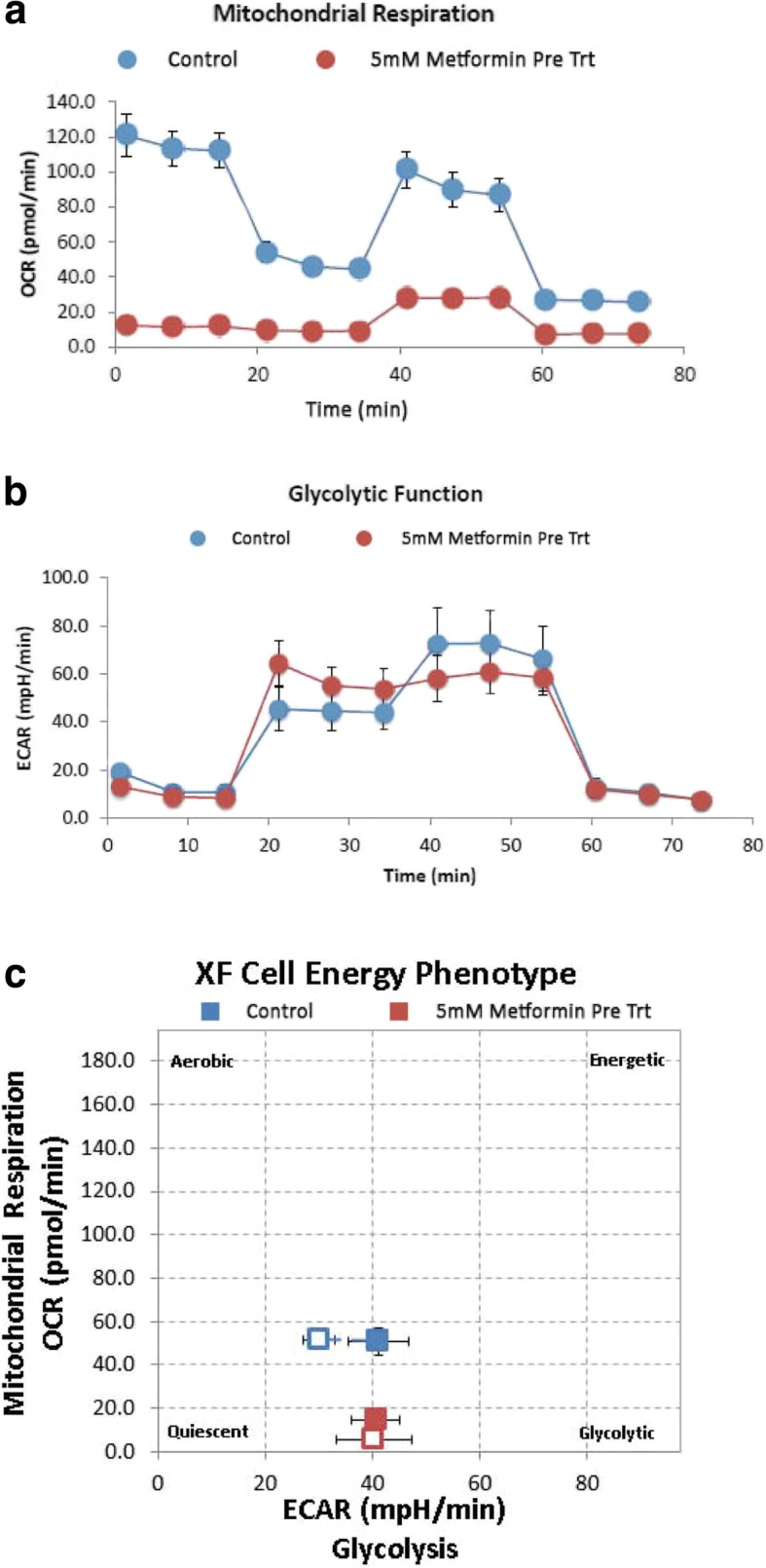
**a** OXPHOS metabolism associated with metformin treatment. **b** glycolysis after metformin treatment and **c** metabolic phenotype. All analysis were performed after 18 h treatment with 5 mM of metformin. Red line is metformin and blue line media control

MP1 at concentrations of 0.20 μM had inhibitory effects on OXPHOS metabolism. There were major effects on maximal respiration rate in the analysis of OXPHOS after overnight treatment (18 h) with MP1 with an accompanied drop in ATP. There was a compensatory rise in glycolysis, presumably as an initial stress response in order to maintain ATP levels that also trended towards statistical significance (Fig. 3a). Treatment with MP1 at a concentration of 0.50 μM resulted in a near complete uncoupling of OXPHOS and inhibition of glycolysis. This complete inhibition of metabolism led to a severe decline in ATP production (Fig. 3b). There was a non-statistically significant drop in OXPHOS metabolism and stimulation of glycolysis at an MP1 concentration of 0.1 μM corresponding to the IC_50_ of 0.096 μM in the MTT assay (Additional file 3: Figure S3a and b). Using a metabolic phenotyping assay overnight treatment (18 h) with MP1 led to a quiescent phenotype that was concentration related (Fig. 4a,b,c,d). In comparison, metformin (5 mM) which is a metabolic inhibitor proposed for use in various cancers, uncoupled OXPHOS metabolism without effects on glycolysis, a phenotype consistent with maintenance of Warburg metabolism (Fig. 5a,b,c). These effects of metformin were concentration dependent similar to MP1 but at concentrations more than 1000 times higher than those of MP1.

### Electron microscopy (EM) of BE-2c with and without MP1 treatment

**Fig. 6:**
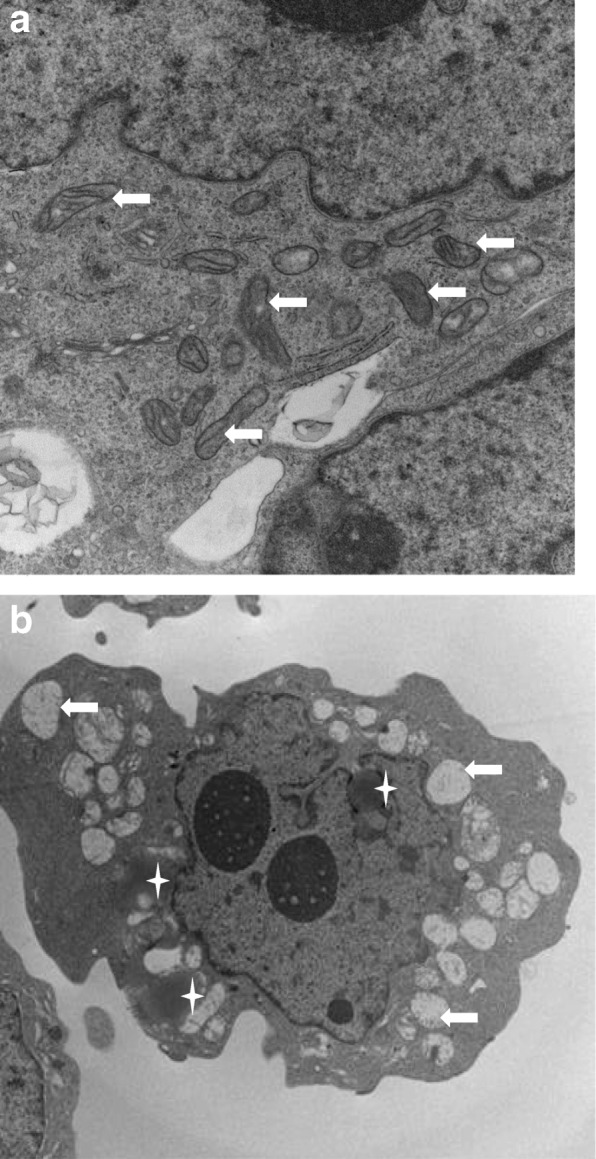
Transmission electron micrographs of BE-2c cells with arrows showing mitochondria; **a** controls with well-defined cristae and the typical elongated mitochondria **b** treatment with MP1 at 0.5 μM show mitochondria with cristae loss and a rounded up morphology. Asterisks identify lipid dense vesicles

Compared to DMSO, MP1 treated cells (0.5 μM) had disruption of mitochondria with loss of cristae and a change in morphology from an elongated morphology to a more rounded morphology. Other observations after MP1 treatment include an increase in double membrane intracellular vesicles which were interpreted as autophagosomes, increased lipid vesicles, increased myelin bodies, and disruption of the cell membrane. There was no indication of major effects on nuclear morphology. Figure 6a and b shows the predominant effect of MP1 on EM, a change in mitochondrial morphology and loss of cristae.

### Western blots for MYCN and MCL-1 (oncogenes), cleaved PARP, bcl-2, BAX (apoptosis/necrosis), BRD-4, and LC3I and LC3II (autophagy)

**Fig. 7:**
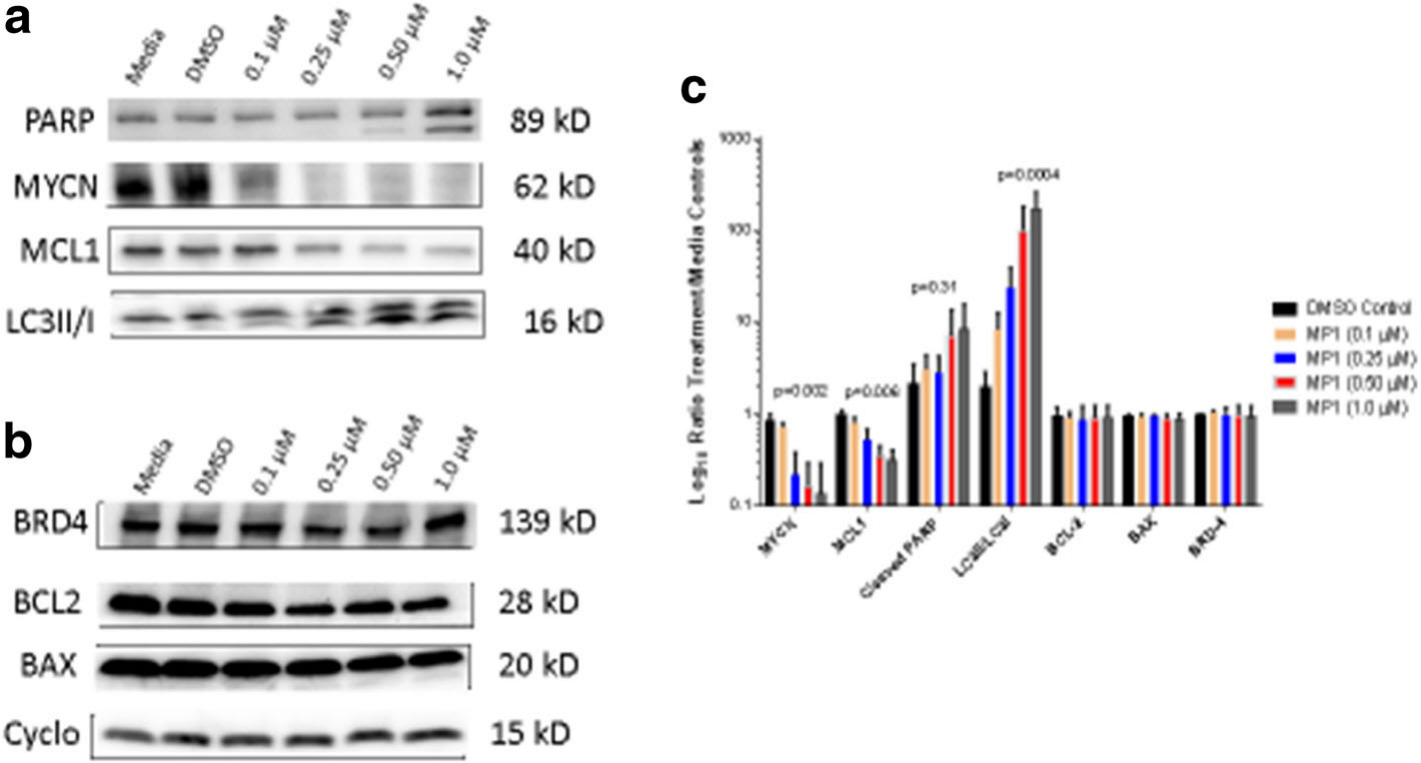
**a** MYCN western blot after 18 h of treatment with increasing concentrations of MP1 (0.1 μM, 0.25 μM, 0.50 μM, 1.0 μM) in BE-2c cells. Increased cleaved PARP indicative of apoptosis/necrosis, decreased MCL-1 and MYCN protein expression, and increased LC3II (lower band) indicative of stimulation of autophagy with all values normalized using total protein. **b** BRD-4, BCL2, and BAX protein reported after normalization using cyclophilin loading controls. MYCN reported as mean ± SD of 6 experiments, MCL-1 as mean ± SD of 4 experiments, cleaved PARP as mean ± SD of 3 experiments, and LC3II/LC3I as mean ± SD of 3 experiments. BRD4, BCL2, and BAX reported as mean ± SD of 3 experiments **c** Ratio of treatment (DMSO control, MP1 0.1 µM, 0.25 µM, 0.5 µM, and 1.0 µM) to media control for MYCN, MCL-1, cleaved PARP, LC3II/I, bcl-2, BAX, and BRD-4 proteins

Western blots for proteins involved in apoptosis, autophagy and survival were performed. MYCN was primarily evaluated because of its central role as an oncogene and potential therapeutic target in high-risk NB. A time course indicated that MYCN expression declined significantly 18–24 h after MP1 treatment (data not shown). Given that the effects of MP1 on inhibition of MYCN were maximal at 18–24 h, the majority of the subsequent experiments were performed after 18 h of MP1 treatment. The decline in MYCN and MCL-1, and increase in LCII protein were all statistically significant while the increase in cleaved PARP was not statistically significant (Fig. 7a,c). BRD-4, bcl-2, and bax expression was not significantly changed by MP1 treatment (Fig. 7b,c).

### Gene expression for MYCN, MCL-1, PARP, LC3I and LC3II, BRD-4, bcl-2, and BAX

RNAseq results shown in Table 2 generally supported the changes seen in protein expression with a decline, by a factor of two, for MYCN gene expression after MP1 treatment but only a minor decline in MCL-1 (− 1.18x). The corresponding protein expression indicated a near complete loss of MYCN and a diminished but still apparent MCL-1 band. LCII gene expression was increased after MP1 treatment (+ 1.8) compared to control. LCI was also increased but to a lesser extent (+ 1.6). This data was also consistent with western blots which showed an increase in LCII to LCI ratios. There was a decline in PARP-1 gene expression (− 1.5x) and an increase in protein cleavage after MP-1 treatment. BRD-4 gene expression was unchanged while bcl-2 increased (1.76x) and BAX decreased (− 1.39x). None of these effects on gene expression led to significant changes in protein expression. Changes in gene expression of 1.6–2.0 times or greater have been considered biologically significant but smaller changes may be important [15]. Highly statistically significant changes in gene expression were seen with MYCN, MCL-1, PARP, LC3, bcl-2, and BAX with only MYCN, LC, and bcl-2 genes meeting that criteria of a factor of 1.6 fold change or greater.

**Table 2:** RNAseq comparing no treatment controls versus 18 h treatment with: a.) control versus MP1 at 500 nM, b.) control versus TEM alone at 1 μM, and c) control versus combination of MP1 plus TEM. All treatments were performed in triplicate and fold change calculated from the ratio of treatment over control

a.)								
Gene	Change	*p*-value	Cntrl#1	Cntrl#2	Cntrl #3	MP1#1	MP1#2	MP1#3
MYCN	−2.0	6.09 E-179	2125	2166	2202	1092	1087	1096
MCL-1	−1.18	3.38E-09	318	309	315	265	268	267
LCII	+ 1.8	4.08 E-59	67.5	70.7	76.0	135	128.9	131.4
LCI	+ 1.6	1.38 E-03	2.68	3.8	2.67	4.23	6.13	4.27
PARP1	−1.5	8.56 E-52	699	711.6	689.6	451.8	466.3	463.9
BRD-4	+ 1.07	0.15	77.3	75.5	75.2	84.5	82.2	77.4
Bcl-2	1.76	1.09 E-35	33.7	34.6	34.1	64.0	50.5	56.9
BAX	−1.39	8.51 E-12	46.1	47.6	43.4	35.0	31.5	32.0
b.)								
Gene	Change	*p*-value	Cntrl#1	Cntrl #2	Cntrl #3	TEM#1	TEM#2	TEM#3
MYCN	1.0	0.35	2125	2166	2202	2264	2140	2232
MCL-1	−1.12	2.48E-05	318	309	315	281	282	276
LCII	+ 1.3	1.44 E-10	67.5	70.7	76.0	92.7	92.5	89.7
LCI	+ 1.5	0.004	2.68	3.8	2.67	5.1	4.4	4.4
PARP1	−1.2	1.01E-08	699	712	690	580	617	599
BRD-4	−1.06	0.20	77.3	75.5	75.2	70.4	71.4	72.7
Bcl-2	1.45	9.03 E-16	33.7	34.6	34.1	49.2	52.0	47.7
BAX	−1.14	0.007	46.1	47.6	43.4	42.7	37.3	40.6
c.)								
Gene	Change	*p*-value	Cntrl#1	Cntrl #2	Cntrl #3	MP1 + TEM #1	MP1 + TEM #2	MP1 + TEM #3
MYCN	−1.6	9.1E-89	2125	2166	2202	1338	1347	1356
MCL-1	−1.2	1.29E-08	318	309	315	266	271	269
LCII	+ 1.7	2.28 E-42	67.5	70.7	76.0	113	125	120
LCI	+ 2.5	2.11E11	2.68	3.8	2.67	8.19	8.21	6.5
PARP1	−1.6	6.48E-64	699	712	690	432	440	447
BRD-4	−1.09	0.078	77.3	75.5	75.2	78.6	62.5	68.8
Bcl-2	1.94	5.9 E-51	33.7	34.6	34.1	66.2	63.8	68.5
BAX	−1.29	8.67 E-08	46.1	47.6	43.4	34.3	36.5	35.8

### In-vivo activity, toxicity, and concentrations of MP1 in blood, organs and tumor

**Fig. 8:**
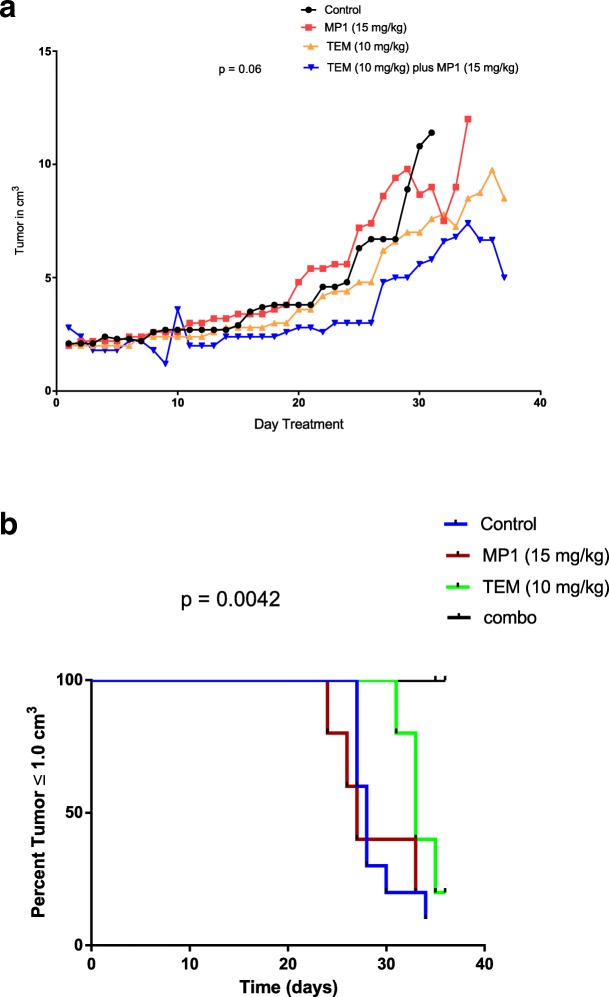
**a** Comparison of diluent controls (*N* = 10) to MP1 alone (*N* = 5), TEM alone (*N* = 5), and the combination (*N* = 5). Each group injected with 5 × 10^5^ cells subcutaneously with each time-point graphed as mean tumor size. **b** Kaplan-Meier Analysis of the time to development of tumor of 1 cm^3^. Statistical significance between tumor growth rate curves determined using ANOVA and statistical significance between groups on time to ≥1cm^3^ tumor determined using a log-rank test. Statistical significance was defined at *p* ≤ 0.05

A bio-distribution study in 6 tumor bearing mice orally dosed with MP1 was performed to determine MP1 concentrations which could be achieved in-vivo including in plasma, liver, lungs, spleen, brain, and tumor. While there was variability in MP1 concentrations in tumor, concentrations above the IC_50_ were achieved (Table 3). MP1 alone appeared to be well tolerated with no deaths. The bio-distribution study was followed by a pilot study of TEM plus MP1 to investigate anti-tumor activity in 5 mice. The combination was selected based on in-vitro studies demonstrating potentiation. Three mice of the five had tumor regression with tumors going from palpable tumors (2mm^3^) to non-palpable tumors. Resection of the tumor site in the three mice obtaining a complete response found no tumor in one of the three with non-palpable residual disease in the other two. MP1 was well tolerated with bone marrow unaffected by TEM alone, MP1 alone, or the combination compared to untreated controls as determined by a CFU-GM assay (Additional file 4: Figure S4). There was also no weight loss during the course of the study (data not shown). A third experiment compared diluent controls (*N* = 10) to MP1 alone (*N* = 5), TEM alone (*N* = 5), and the combination (*N* = 5). The combination group showed a decline in tumor growth rates compared to the other groups which approached significance (*p* = 0.06) (Fig. 8) without weight loss (Additional file 5: Figure S5). There were no complete responses in any of the four groups.

**Table 3:** Plasma, organ, and tumor concentrations of MP1 in NSG mice dosed with 15 mg/kg MP1 five times per week orally. Samples collected at necropsy at 30 min or 24 h after dosing of MP1

Mouse ID	Time of collection	Plasma Conc (ng/mL)	Liver Conc (ng/g)	Lungs Conc (ng/g)	Tumor Conc (ng/g)	Spleen Conc (ng/g)	Fore Brain Conc (ng/g)	Mid Brain Conc (ng/g)	Hind Brain Conc (ng/g)
BE-326	30 min	571.2	4122.2	2154.6	1433.0	AB	52.0	167.3	72.6
BE-329	30 min	244.6	2235.1	1053.2	2156.7	279.7	39.3	30.8	34.5
BE-330	30 min	719.1	2627.7	1719.7	796.6	461.2	56.1	57.8	58.5
BE-327	24 h	6.7	41.8	14.5	3.8	AB	0.5	0.6	0.7
BE-331	24 h	12.2	30.6	11.9	2.3	AB	10.7	13.8	22.7
BE-332	24 h	38.1	46.7	5.5	1.7	1.2	0.2	0.1	0.3

## Discussion

NB is the most common extracranial solid tumor occurring in children and the treatment of metastatic disease continues to be challenging. Especially problematic is the treatment of children with high-risk disease, who have survival rates of less than 40% at 5 years, despite aggressive multimodal therapy, with mortality resulting from relapsed chemoresistant disease [16]. Therefore, innovative approaches to the treatment of relapsed/refractory NB are needed.

The marinopyrrole MP1 demonstrated potent activity in a chemoresistant MYCN amplified NB cell line, BE-2c cells. The proposed mechanism of the marinopyrroles is the inhibition of MCL-1 interaction with its binding partner Bim [17]. Given that MCL-1 is commonly over-expressed in many cancers, including neuroblastoma, MCL-1 is an attractive target, particularly in combination with other therapies, including chemotherapy and other targeted therapies [18, 19]. The activity of MP1 in BE-2c cell culture suggests that MYCN might be an important target in addition to MCL-1.

MYC is an oncogene commonly amplified in a wide variety of tumors [20]. More specifically MYCN is an oncogene and tumor driver in high-risk poor prognostic NB [21]. While marinopyrrole inhibition of MCL-1 has been described previously, inhibition of the MYCN oncogene has not been reported. In the initial screening experiments with MP1 against three NB cell lines, the extent of activity correlated with the magnitude of MYCN amplification. Additional support for MP1 targeting of MYCN was a decline in MYCN expression both at the mRNA and protein levels. The effect on MCL-1 expression was more modest and unlikely to explain the activity of MP1 in BE-2c cells. These data imply that for the MP1 analog, MYCN and not MCL-1, is the predominant target. Whether this is a direct antagonism based on directly binding to MYCN or an indirect effect is an active area of investigation. It does appear that the inhibition of the epigenetic regulator BRD-4 is not the mechanism by which MP1 inhibits MYCN given the lack of effect on gene or protein expression. This is an important observation given the early clinical use of BRD-4 inhibitors in MYCN amplified NB [22].

The effect of MYC inhibition has previously been reported to lead to the accumulation of intracellular lipid and a similar effect was seen on EM in this study [23]. A more quantitative evaluation of metabolism showed that MP1 treatment in-vitro produced major disruption of glycolysis and OXPHOS metabolism, likely from the inhibitory effects on MYCN pathways. The effect on OXPHOS occurred at nanomolar concentrations. Inhibition of glycolysis occurred at higher nanomolar concentrations which remain achievable in-vivo. Disruption of mitochondrial function was evident using metabolic flux analysis where mitochondrial polarization and ATP production had major declines after treatment with MP1. It has been previously reported that amplification of MYCN increases OXPHOS metabolism and inhibition of MYCN leads to disruption of OXPHOS which mirrors our results. It is interesting that glycolysis is inhibited as well by MP1 which is distinct from the metabolic inhibitor metformin. A direct effect on mitochondria is likely since mitochondrial ultrastructure was severely disrupted after MP1 treatment. The cristae disruption and rounding-up of mitochondria in MP1 treated cells has been reported to be associated with a decline in OXPHOS metabolism and ATP production and may be associated with an elevation in reactive oxygen species [24].

Metformin is a well-studied metabolic inhibitor which has been investigated as an anti-cancer agent. Its major metabolic effect is on OXPHOS metabolism via inhibition of Complex 1 of the mitochondrial electron transport system [25, 26]. The relevance of this effect has been debated since it is seen at concentrations in the millimolar range well above those that can be obtained in-vivo. As a validation step for the use of BE-2c cells as a model for metabolic modulators, we confirmed the metabolic effect of metformin at a concentration of 5 mM. (Fig. 5). Metformin effects on metabolism were used to compare and contrast with MP1. MP1 was much more potent and produced a more complete inhibition of metabolism compared to metformin in BE-2c cells. Whether these attributes of MP1 will be more useful in a clinical setting is unknown and will require continued development, but MP1 and similar marinopyrroles may be an improvement on metformin which has generally had modest success in the prevention and treatment of cancer [27]. In addition to its effects on mitochondria and OXPHOS metabolism metformin may have effects on PI3K/Akt/mTor pathways and AMPK. Whether MP1 also has effects on these targets is a active area of investigation [28].

The drop in ATP production associated with MP1 treatment corresponded to a stimulation of autophagy as suggested by an increase in LC3II/LC3I ratios [29, 30]. Metabolic phenotyping showed that MP1 induced quiescence. Because apoptosis is energy requiring, the drop in cellular ATP may favor necrotic cell death over apoptosis and is compatible with EM observations where cell membrane disruption and cell lysis was observed after MP1 treatment without effects on nuclear morphology [31, 32]. On the other hand there was a non-significant but moderate increase in cleaved PARP at higher concentrations of MP1 suggesting a role for apoptosis [33]. However, this was not supported by changes in the pro-apoptotic BAX protein.

The uncoupling of OXPHOS metabolism and disruption of the internal structure of mitochondria seen on EM support the mitochondria as a target of MP1 in MYCN amplified NB. It has been suggested that targeting the mitochondria and metabolism may lead to unacceptable toxicity which has limited the enthusiasm for the potential translation of mitochondrial targeting compounds in cancer [34]. However, recent targeting of mitochondria in cancer has not demonstrated overt or histologic evidence of toxicity including in the heart which might be particularly sensitive to associated drops in ATP [35]. Differences in the mitochondria of cancer cells compared to normal cells, including more anionic membrane potential, mtDNA defects, distinctive transporters, and a distinct bioenergetic phenotype are increasingly considered exploitable targets in cancer treatment [35, 36]. Data presented in this report including major disruption of mitochondrial ultrastructure and morphology and a severe drop in OXPHOS metabolism produced little evidence of toxicity in mice. Mice maintained their weights and showed no evidence of behavioral changes associated with dosing of MP1. This tolerability study was limited by its short duration and small numbers of mice. A subsequent efficacy study also supported an acceptable toxicity profile with mice maintaining baseline weights without any mortality over the 30 days of the study. In addition, there was no effect on bone marrow function a common toxicity of most anti-cancer therapies.

The addition to TEM to MP1 was a reasonable strategy based on the activation of TORC1 pathways in MYC amplified cancers. In addition, TEM has recently been included in clinical trials for relapsed/refractory NB. The tumor regression seen in this study in NSG mice treated with a combination of MP1 and TEM is proof of principle for this combination. It also more generally supports the use of metabolic inhibitors such as TEM and MP1 in combination in MYC amplified cancers [37, 38]. TEM was chosen as the treatment to pair with MP1 in-vivo based on high activity of this combination in-vitro and the current use of TEM in clinical trials for NB [39, 40]. A similar process will be used to test other combinations including in retinoic acid and proteasome inhibitors.

## Conclusions

MP1 belongs to a new class of anticancer compounds, the marinopyrroles and their derivatives, pyrrolomycins, which have shown promising in-vitro activity in MYCN amplified highly resistant NB. MP1 inhibited MYCN and MCL-1 expression and stimulated autophagy consistent with observed treatment related inhibition of OXPHOS metabolism and depletion of cellular ATP. These metabolic effects led to a quiescent phenotype as evidenced by metabolic decline in oxygen utilization. In comparison to metformin, MP1 created a much more potent and complete metabolic inhibition having effects on both OXPHOS and glycolysis as opposed to OXPHOS alone. Anti-tumor activity of MP1 in cell culture and in tumor bearing mice was potentiated by TEM, by mechanisms that likely involve the mTOR pathway.

## Additional files

Additional file 1: **Figure S1.** a.) Activity of MP1 in MYCN non-amplified SKN-AS NB cells and b.) activity of MP1 alone in moderately MYCN amplified IMR-32 cells. MP1 after 18 h treatments at concentrations of 0.1 μM, 0.25 μM, 0.5 μM, 1.0 μM, 5.0 μM, 10 μM, and 50 μM of MP1. Reported as mean of 8 values plus/minus SD. (DOCX 491 kb)

Additional file 2: **Figure S2.** ddPCR showing MYCN gene copy number adjusted for normal diploid reference gene, N-acetylglucosamine kinase (NAGK). (DOCX 27 kb)

Additional file 3: **Figure S3.** Treatment of BE-2c cells with MP1 at 0.1 μM: a) mitochondrial stress test shows a non-statistically significant inhibition on OXPHOS and b) non-statistical significant stimulation of glycolysis. (DOCX 365 kb)

Additional file 4: **Figure S4.** Effect of TEM, MP1, and the combination on bone marrow colony forming unit (CFU) compared to controls. No statistically significant difference between groups. (DOCX 16 kb)

Additional file 5: **Figure S5.** Mouse Weights in control, MP1 alone (*N* = 10), TEM alone (*N* = 5), MP1 alone (*N* = 5) and the combination (*N* = 5). No statistically significant difference between group. (DOCX 44 kb)

## Data Availability

No large data are included in this manuscript. Cell line verification was performed for BE-2c, IMR, and SKN-AS using STR profiling through ATCC and is available upon request.

## Abbreviations

DMEM: Dulbecco’s Modified Eagle Media
DMSO: Dimethyl Sulfoxide
ECAR: Extracellular acidification rate
EMEM: Eagle’s Minimum Essential Medium
IACUC: Institutional Animal Care and Use Committee
MTT: 3-(4,5-Dimethylthiazol-2yl)-2,5-Diphenyltetrazolium
NSG: NOD-scid Gamma Immunodeficient Mice
OCR: Oxygen consumption rate
OXPHOS: Oxidative phosphorylation

## References

[1] Ward E, DeSantis C, Robbins A, Kohler B, Jemal A (2014). Childhood and adolescent cancer statistics, 2014. CA Cancer J Clin.

[2] Hallett A, Traunecker H (2012). A review and update on neuroblastoma. Paediatr Child Health.

[3] Aminzadeh S, Vidali S, Sperl W, Kofler B, Feichtinger RG (2015). Energy metabolism in neuroblastoma and Wilms tumor. Transl Pediatr.

[4] Doherty JR, Cleveland JL (2013). Targeting lactate metabolism for cancer therapeutics. J Clin Invest.

[5] Chourasia AH, Boland ML, Macleod KF (2015). Mitophagy and cancer. Cancer Metabol.

[6] Dejure F, Eilers M (2017). MYC and tumor metabolism: chicken and egg. EMBO J.

[7] Chandel N (2014). Four key questions about metformin and cancer. BMC Biol.

[8] Li R (2016). Marinopyrroles: unique drug discoveries based on marine natural products. Med Res Rev.

[9] Li R, Sebti SM, Liu Y, Qin Y, Song H, Cheng C. Marinopyrrole derivatives and methods of making and using same. U.S. patent no. 2018;9,868,474.

[10] Li R, Sebti SM, Liu Y. Marinopyrrole derivatives as anticancer agents. U.S. patent no. 2016;9,340,501.

[11] Cheng C, Pan L, Chen Y, Song H, Qin Y, Li R (2010). Total synthesis of (±) marinopyrrole A and its library as potential antibiotic and anticancer agents. J Comb Chem.

[12] Doi K, Li R, Sung SS, Wu H, Liu Y, Manieri W (2012). Discovery of marinopyrrole A (Maritoclax) as a selective Mcl-1 antagonist that overcomes ABT-737 resistance by binding to and targeting Mcl-1 for proteasome degradation. J Biol Chem.

[13] Thiele CJ, Masters J (1998). Neuroblastoma. Human cell culture.

[14] Lodrini M, Sprussel A, Astrahantseff K, Tiburius D, Konschak R, Lode H (2017). Using droplet digital PCR to analyze MYCN and ALK copy number in plasma from patients with neuroblastoma. Oncotarget.

[15] St. Laurent G, Shtokalo D, Tackett M, Yang Z, Vyatkin Y, Milos P (2013). On the importance of small changes in RNA expression. Methods.

[16] Shohet J, Foster J (2017). Neuroblastoma. Br Med J.

[17] Li R, Cheng C, Balasis ME, Liu Y, Garner TP, Daniel KG (2015). Design, synthesis, and evaluation of marinopyrrole derivatives as selective inhibitors of mcl-1 binding to pro-apoptotic Bim and dual Mcl-1/Bcl-xL inhibitors. Eur J Med Chem.

[18] Perciavalle R, Stewart D, Koss B, Lynch J, Milasta S, Bathina M (2012). Anti-apoptotic MCL-1 localizes to the mitochondrial matrix and couples mitochondrial fusion to respiration. Nat Cell Biol.

[19] Lestini BJ, Goldsmith KC, Fluchel MN, Liu X, Chen NL, Goyal B (2009). MCL-1 downregulation sensitizes neuroblastoma to cytotoxic chemotherapy and small molecule BCL-2 family antagonists. Cancer Biol Ther.

[20] Rickman D, Schulte J, Eilers M (2018). The expanding world of NMYC driven tumors. Cancer Discov.

[21] Ham J, Costa C, Sano R, Lochmann T, Sennott E, Patel (2016). Exploitation of the apoptosis-primed state of MYCN-amplified neuroblastoma to develop a potent and specific targeted therapy combination. Cancer Cell.

[22] Felgenauer J, Tomino L, Anderson JS, Bopp E, Shah N (2018). Dual BRD4 and AURKA inhibition is synergistic against MYCN amplified and non-amplified neuroblastoma. Neoplasia.

[23] Zirath H, Frenzel A, Oliynyk G, Segerstrom L, Westermark UK, Larsson K (2013). MYC inhibition induces metabolic changes leading to accumulation of lipid droplets in tumor cells. PNAS.

[24] Cogliati S, Enriquez J, Scorrano L (2016). Mitochondrial cristae: where beauty meets functionality. Trends Biochem Sci.

[25] Daugan M, Wojcicki A, Hayer B, Boudy V (2016). Metformin: an anti-diabetic drug to fight cancer. Pharmacol Res.

[26] Zi F, Zi H, Li Y, He J, Shi Q, Cai Z (2018). Metformin and cancer: an existing drug for cancer prevention and therapy. Oncol Lett.

[27] Vancura A, Bu P, Bhagwat M, Zeng J, Vancurova I (2018). Metformin as an anticancer agent. Trend Pharm Sci.

[28] Rizos C, Elisaf M (2013). Metformin and Cancer. Eur J Pharmacol.

[29] Lee J, Giordano S, Zhang J (2012). Autophagy, mitochondria and oxidative stress: cross-talk and redox signaling. Biochem J.

[30] Barth S, Glick D, Macleod K (2010). Autophagy: assays and artifacts. J Pathol.

[31] Moriwaki K, Chan F (2013). RIP3: a molecular switch for necrosis and inflammation. Genes Dev.

[32] Eguchi Y, Shimizu S, Tsujimoto Y (1997). Intracellular ATP levels determine cell death fate by apoptosis or necrosis. Cancer Res.

[33] Shin H, Kwon H, Lee J, Gui X, Achek A, Kim J (2015). Doxorubicin-induced necrosis is mediated by poly-(ADP-ribose) polymerase 1 (PARP1) but is independent of p53. Sci Rep.

[34] Dykens J, Will Y (2007). The significance of mitochondrial toxicity testing in drug development. Drug Dis Today.

[35] Leanza L, Romio M, Becker K, Azzolini M, Trentin L, Manago A (2017). Direct pharmacological targeting of a mitochondrial ion channel selectively kills tumor cells in vivo. Cancer Cell.

[36] Wallace DC (2012). Mitochondria and cancer. Nat Rev Cancer.

[37] Polivka J, Janku F (2014). Molecular targets for cancer therapy in the PI3K/AKT/mTOR pathway. Pharmacol Ther.

[38] Vaughn L, Clarke P, Barker K, Chanthery Y, Gustafson C, Tucker E (2016). Inhibition of mTOR-kinase destabilizes MYCN and is a potential therapy for MYCN-dependent tumors. Oncotarget.

[39] Spunt S, Grupp S, Vik T, Santana V, Greenblatt D, Clancy J (2011). Phase 1 study of temsirolimus in pediatric patients with reccurent/refractory solid tumors. J Clin Oncol.

[40] Geoerger B, Kieran MW, Grupp S, Perek D, Clancy J, Krygowski M (2012). Phase II trial of temsirolimus in children with high-grade glioma, neuroblastoma and rhabdomyosarcoma. Eur J Cancer.

