# Supplementary figures and images for: Effects of novel pyrrolomycin MP1 in MYCN amplified chemoresistant neuroblastoma cell lines alone and combined with temsirolimus
Source: BMC Cancer. 2019 Aug 27;19:837. doi: 10.1186/s12885-019-6033-2 (PMC6712804; doi:10.1186/s12885-019-6033-2)

Supplemental Figure 3.

a.


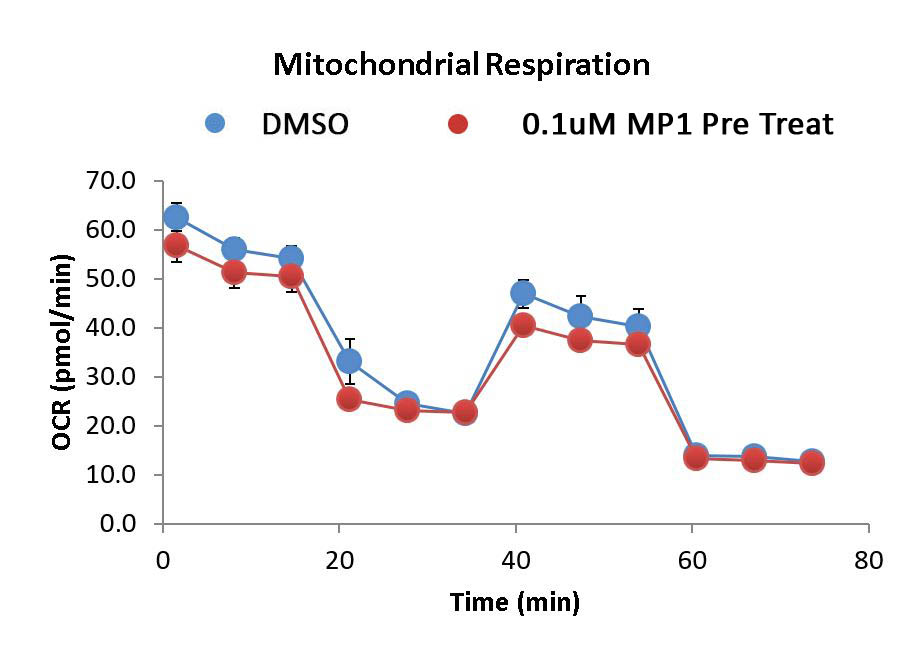


**p = 0.06**

b.


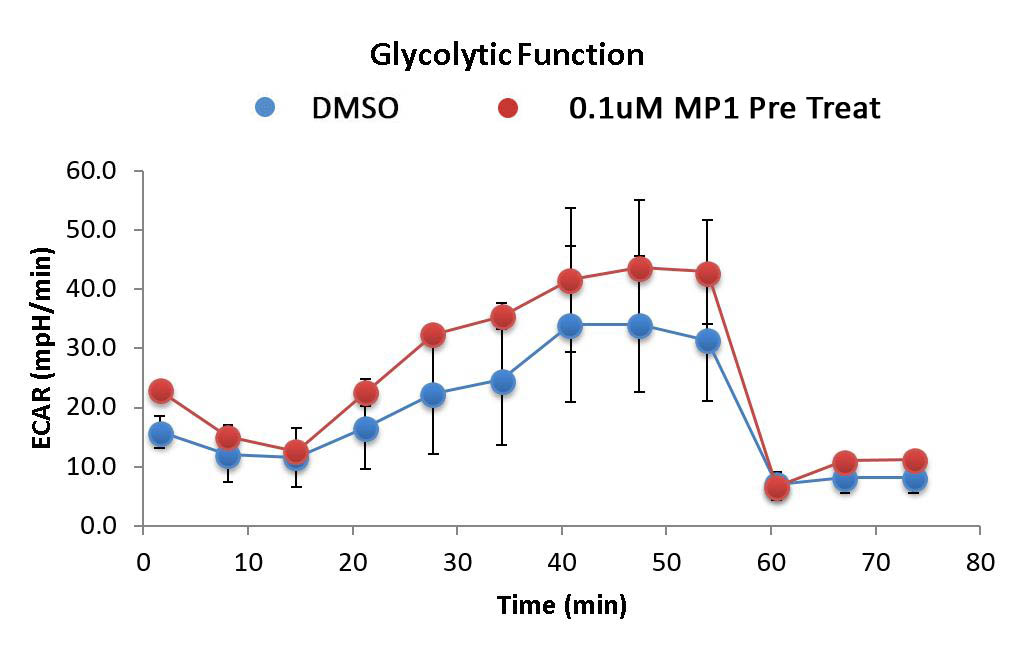


**p = 0.16**

Supplement: Supplementary file 3 — Figure S3. Treatment of BE-2c cells with MP1 at 0.1 μM: a) mitochondrial stress test shows a non-statistically significant inhibition on OXPHOS and b) non-statistical significant stimulation of glycolysis. (DOCX 365 kb) [file 12885_2019_6033_MOESM3_ESM.docx]
